# Exodontia associated bacteremia in horses characterized by next generation sequencing

**DOI:** 10.1038/s41598-021-85484-z

**Published:** 2021-03-18

**Authors:** Kile S. Townsend, Philip J. Johnson, Alison M. LaCarrubba, Lynn M. Martin, Aaron C. Ericsson

**Affiliations:** 1 Department of Veterinary Medicine and Surgery, College of Veterinary Medicine, University of Missouri, Columbia, MO 65211 USA; 2 Department of Veterinary Pathobiology, College of Veterinary Medicine, University of Missouri, Columbia, MO 65211 USA

**Keywords:** Microbiology, Bacteria, Bacteriology, Dental diseases

## Abstract

Bacteremia resulting from dental surgery is increasingly recognized as a health risk, especially in older and immunocompromised patients. Dentistry-associated bacteremia can lead to remote infections, as exemplified by valvular endocarditis. Emerging evidence points to a novel role played by oral cavity commensals in the pathogenesis of diabetes, respiratory disease, cardiovascular disease, and adverse pregnancy outcomes. Whether dental extraction, a commonly undertaken procedure in old horses, causes bacteremia has not been reported extensively. In a prospective clinical study using next generation sequencing (based on bacterial 16S rRNA), the circulating blood microbiome was characterized before and at 1 h following extraction of incisor, canine or cheek teeth from 29 adult horses with dental disease. 16S rRNA gene sequencing results from the blood microbiome were compared with those from gingival swab samples obtained prior to extraction at the location of the diseased tooth. Bacteremia associated with translocated gingival commensals was demonstrated in horses undergoing exodontia and was, in some cases, still evident one hour post-operatively.

## Introduction

Exodontia (tooth extraction) and periodontal disease are associated with bacteremia, a phenomenon that may lead to adverse health outcomes^1–12^. Numerous reports over the last 50 years have documented the pathophysiological association between periodontal disease and systemic conditions such as diabetes mellitus, atherosclerotic cardiovascular disease, metabolic syndrome, chronic diseases (i.e., rheumatoid arthritis, cancer, Alzheimer’s disease), and adverse pregnancy outcomes^1,3,4,8,13^. Whereas any relationship between exodontia-associated bacteremia and the number of extracted teeth is presently unclear, it is associated with concurrent odontogenic infection in affected people^10^. Post-exodontia bacteremia has also been associated with infection at distant sites such as the heart (valvular endocarditis), respiratory tract, joints, and brain^4,14^.

Recently, veterinarians have also recognized associations between periodontal disease and systemic health in canine and feline species^15–17^, leading to broader recognition of the importance of routine oral examination and preventive dentistry in veterinary clinical practice. Additionally, the extent to which veterinarians provide advanced dental care for horses has increased substantially in recent years as a result of parallel increases in both the availability of improved dental surgical equipment and education regarding the technical skills associated with this discipline^18^. Veterinary work with old horses has increased considerably in recent years and clinical problems resulting from age-associated dental attrition represent an especially common component of equine veterinary work in geriatric horses^18–20^. Remarkably, the incidence of periodontal disease in horses has been estimated to be as high as 75%, with the incidence increasing with advancing age^21^.

Dental diseases in horses, such as those associated with equine odontoclastic tooth reabsorption and hypercementosis (EOTRH) syndrome or apical infections, are often unrecognized until relatively late stages, at which time dental extraction is commonly undertaken. Post-exodontia endocarditis, a potential complication of bacteremia, has been described in both dogs and horses^22–24^. Oral cavity microorganisms have been implicated in a few case reports in which fatal bacterial infections (endocarditis, meningitis, and pneumonia) developed following exodontia in horses^25,26^. The extent to which bacteremia leads to numerous complications in other species suggests that its role in equidae may be under-appreciated and deserving of further investigation^4–6,10,14^. These potential complications are important when one considers that exodontia is frequently performed in older horses that are at substantially higher risk of disseminated infection as a result of underlying immune-debilitating comorbidities such as pituitary *pars intermedia* dysfunction (PPID) and immunosenescence^27–30^.

Post-exodontia bacteremia was recently reported by Kern et al. (2017), occurring in 18 out of 20 horses using conventional bacteriological culturing methods^31^. Although this was a noteworthy finding, it was likely limited by the method (conventional microbiological culturing) used to detect blood-borne bacteria, which relies on laboratory cultivation of bacteria present in the circulation. Conventional bacterial culturing is limiting because most oral cavity bacteria are uncultivable^32^. An alternative method, such as 16S rRNA amplicon sequencing allows for identification of not only cultivable bacteria but also bacteria that might be present in small numbers or uncultivable via standard techniques^32–36^.

Therefore, the primary aim of this study was to determine the extent to which dental extraction results in post-procedural bacteremia and to characterize bacterial DNA present in the circulation before and following exodontia in adult horses with dental disease using 16S rRNA amplicon sequencing. A secondary aim was to compare gingival swab microbiomes to blood microbiomes before and following exodontia.

## Results

Of the 29 horses, most were determined to be healthy (n = 24) or affected with paranasal sinusitis (n = 6), PPID (n = 3), or asthma (n = 1). Procedures included those requiring cheek tooth extraction (n = 25) and those requiring incisor or canine tooth extraction (n = 9). Justification for exodontia included: apical tooth root abscessation (n = 9), slab fracture (n = 9), equine odontoclastic tooth reabsorption and hypercementosis (EOTRH) syndrome (n = 8), infundibular caries (n = 5), crown fractures (n = 3), and fractured incisive bone (n = 1). A total of 34 procedures were performed on 29 horses (some horses returned for a second exodontia procedure at least one month after completion of the first). Retropulsion of teeth was needed for extraction in three cases and standard intra-oral tooth extraction was performed in the remaining 31 cases. Sinus lavage was performed post-procedurally in all cases with comorbid sinusitis. Detailed signalment and procedural information are presented in Table 1.

**Table 1:** Table depicting individual horse information including teeth extracted, diagnosis, surgical procedure, and health designation (healthy, affected with PPID, equine asthma, sinusitis).

Patient	Tooth/teeth	Diagnosis	Procedure	Healthy	PPID	Asthma	Sinusitis
Horse 1^a^	111	Slab fracture 111	Removal of 111	x			
Horse 1^b^	110, 211	Recessed and missing parts of crown 110, slab fracture 211	Removal of 110 and removal of palatal slab of 211	x			
Horse 2^a^	Mandibular incisors	EOTRH	Removal of mandibular incisors	x			
Horse 2^b^	Maxillary incisors	EOTRH	Removal of maxillary incisors	x			
Horse 3	109, 209	Nasosinus fistula, sinusitis, apical abscess 109, 209, nasal cyst	Removal of 209, 109, trephination and sinus lavage			x	x
Horse 4	202, 203, 302, 303	EOTRH	Removal of 202, 203, 302, 303		x		
Horse 5	209	Slab fracture 209	Removal of 209	x			
Horse 6	209, 210	Fractured 209, sinusitis, apical abscess 210	Removal of 209, 210, rostral maxillary lavage				x
Horse 7	108, 109	Apical abscessation 108, blunted roots 109	Removal of 108, 109	x			
Horse 8	208, 209	Apical abscessation, sinusitis	Removal of 208, 209, trephanation and sinus lavage				x
Horse 9	204, 404	EOTRH	Removal of 204, 404		x		
Horse 10	Maxillary incisors	EOTRH	Removal of maxillary incisors	x			
Horse 11	209, 210	Slab fracture 209, 210	Retropulsion of 209, 210 and sinus lavage				x
Horse 12	108	Infundibular caries	Removal of 108	x			
Horse 13	108	Sinusitis of the frontal and maxillary sinus (right sided),associated with 108	Removal of 107, 108 caps and sinus lavage				x
Horse 14	310, 410	Slab fractures 310, 410	Attempted retropulsion of 410—failed	x			
Horse 15	111, 211	Slab fractures 111, 211	Removal of 111	x			
Horse 16	107, 108, 209	Apical root abscessation 107, 108, 209	Removal of 107, 108, 209	x			
Horse 17	101, 102, 103	Traumatic fracture of incisive bone and tooth roots	Removal of 101, 102, 103	x			
Horse 18	108	Crown fracture with periodontal disease	Removal of 108	x			
Horse 19	209	Infundibular caries	Removal of 209	x			
Horse 20^a^	Maxillary incisors	EOTRH	Removal of maxillary incisors	x			
Horse 20^b^	308, 409	Apical abscessation 308, 409	Removal of 308, 409		x		
Horse 21^a^	Maxillary incisors	EOTRH	Removal of maxillary incisors	x			
Horse 21^b^	Mandibular incisors	EOTRH	Removal of mandibular incisors	x			
Horse 22	209	Infundibular caries, fracture through pulp horn	Removal of 209	x			
Horse 23	110	Slab fracture, periapical abscess 110	Retropulsion of 110 and sinus lavage				x
Horse 24	109, 209	Apical absessation, periodontal disease 109, 209	Removal of 109, 209	x			
Horse 25	208	Infundibular caries	Removal of 208	x			
Horse 26	206, 306	Complicated crown fracture with pulp exposure 206, fractured fragment of 306	Removal of 206, 306	x			
Horse 27	301	Fractured 301 (suspect traumatic)	Removal of 301	x			
Horse 28^a^	109, 208	Infundibular caries 109, slab fracture 208	Removal of 109	x			
Horse 28^b^	208	Slab fracture	Removal of 208	x			
Horse 29	309, 310	Apical root abscessation 309, 310	Removal of 309, 310	x			

Teeth were numbered using the Modified Triadan System.

^a^refers to the first visit, ^b^refers to second visit.

**Figure 1:**
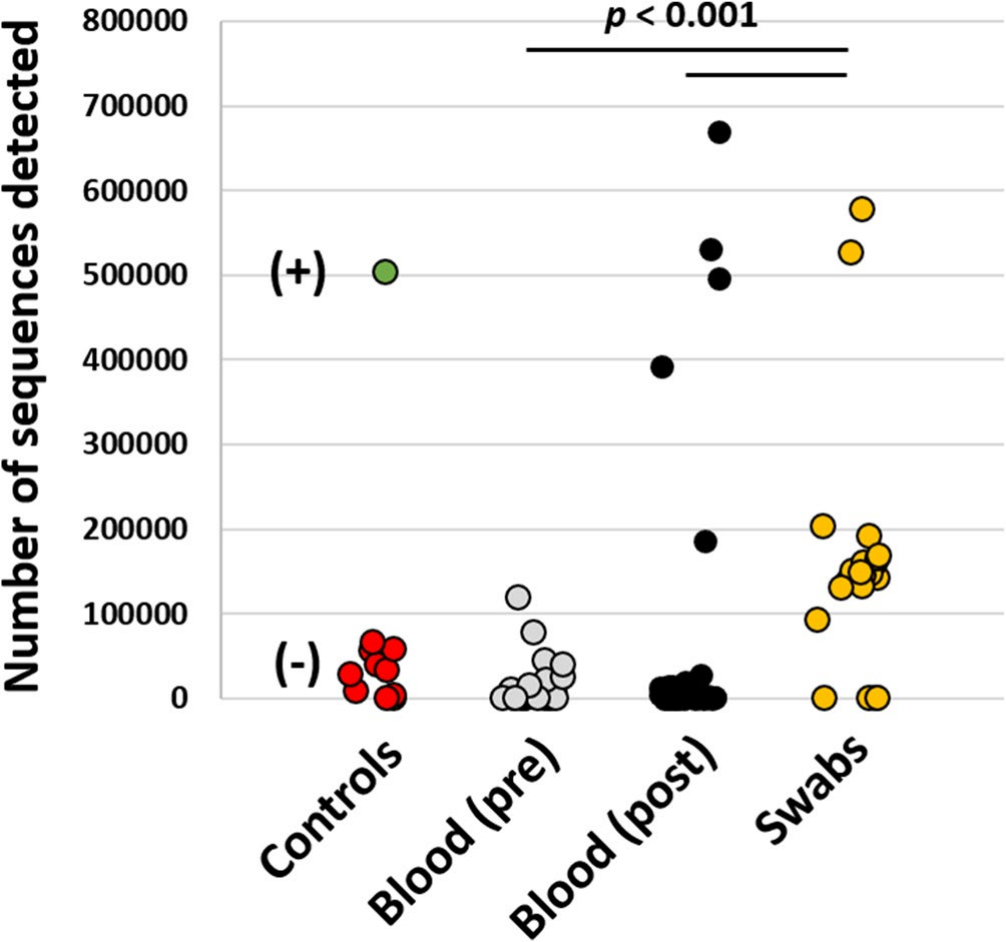
Dot plots showing the total number of 16S rRNA amplicon sequences resulting from amplification and sequencing on a shared flow cell, of negative (−) and positive (+) controls, peripheral blood collected aseptically pre- and post-exodontia procedure, and dental/gingival swabs.

To qualitatively assess the validity of microbial signatures detected via 16S rRNA sequencing, DNA amplification (quantified by total number of reads for a given sample among a shared sequencing flow cell) was compared between pre- and post-exodontia blood samples, gingival swabs, negative reagent controls, and a commercially available bacterial community standard. As anticipated, the swabs yielded higher sequence numbers than either group of blood samples, and the mock community standard yielded higher coverage, by an order of magnitude, than negative reagent controls and most blood samples (Fig. 1). Notably however, five blood samples collected post-exodontia yielded unexpectedly deep coverage, ranging from 187,130 to 669,731 sequences per sample. While sequencing coverage is not absolutely quantitative of starting microbial biomass, these results suggested the presence of increased bacterial biomass in a subset of blood samples collected post-exodontia. Moreover, the validity of the remaining blood samples and a few swabs samples that amplified poorly was brought into question.

**Figure 2:**
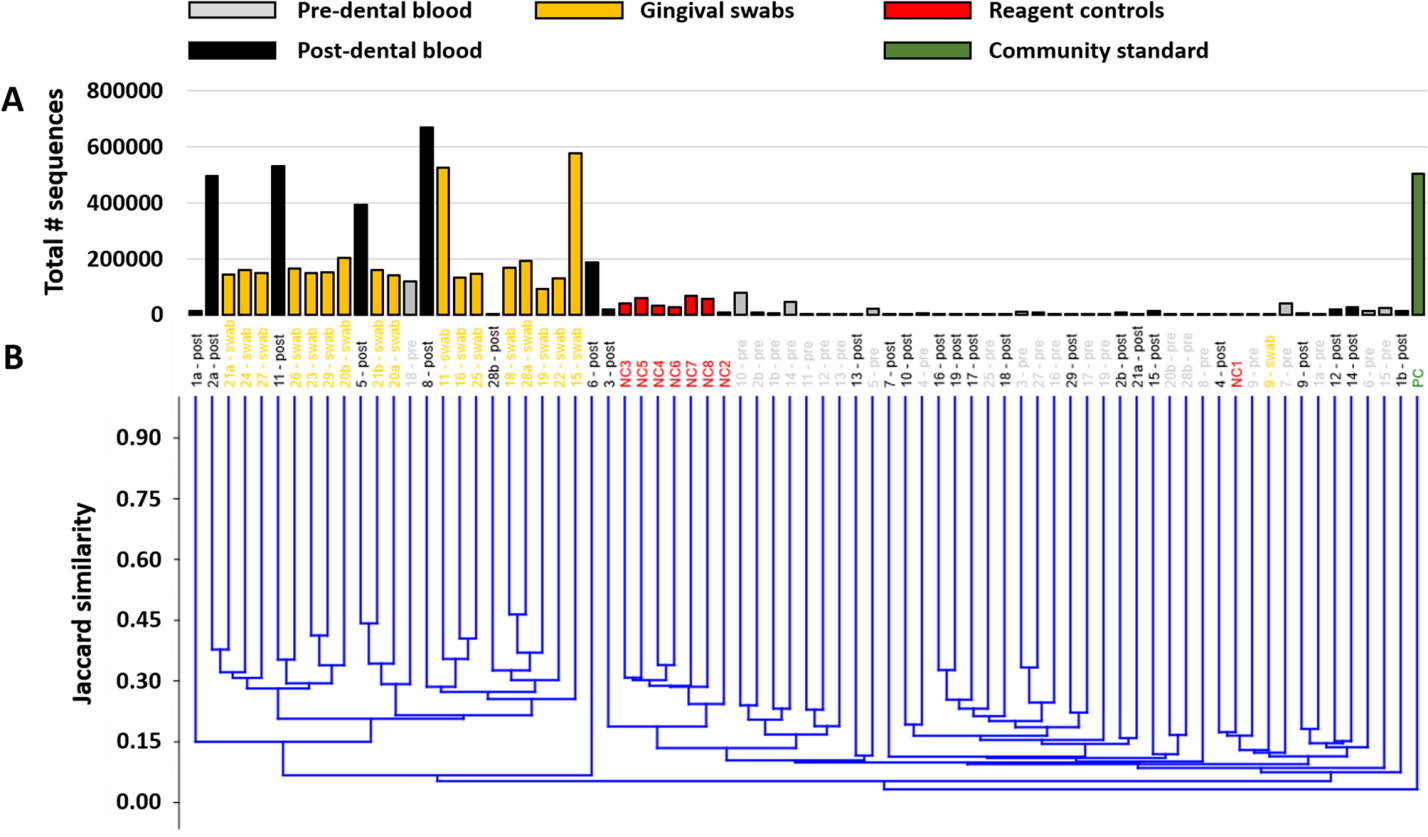
Bar chart showing sample coverage in those samples yielding > 1054 sequences, legend at top (**A**), and a dendrogram generated from those data, rarefied to a uniform coverage of 1054 sequences/sample (**B**).

**Figure 3:**
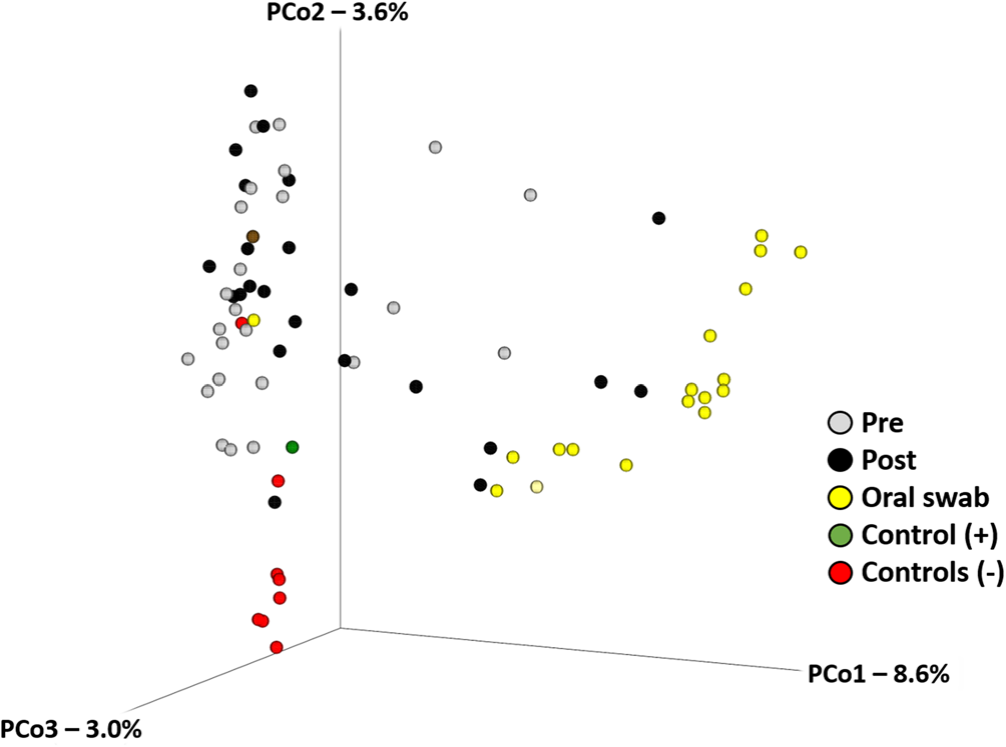
Principal coordinate analysis based on Jaccard similarities and generated using a rarefied dataset (1054 sequences/sample).

Recognizing that the differences in sample coverage would likely skew comparisons of bacterial composition, all samples yielding fewer than 1055 sequences were removed from the following analyses, and the remaining data were rarefied randomly to a uniform read depth of 1054 reads/sample. The original sequencing coverage of those samples (Fig. 2A) is reflective in the hierarchical clustering of samples based on the rarefied dataset, with those same five highly amplified post-exodontia blood samples clustering with the gingival swabs, along with two other post-exodontia and one pre-exodontia blood samples with lower coverage (Fig. 2B). These relationships were also visualized using principal coordinate analysis (PCoA), which demonstrated a similar pattern with the same post-exodontia blood samples clustering close to the gingival swabs (Fig. 3). One-way permutational multivariate ANOVA confirmed significant differences between swabs and pre-exodontia blood (*p* ≤ 0.0001, F = 6.6), swabs and post-exodontia blood (*p* ≤ 0.0001, F = 4.7), and between pre- and post-exodontia blood (*p* = 0.039, F = 1.3). All three groups were significantly different from negative reagent controls (*p* ≤ 0.0001; F = 2.6, 2.6, and 6.2 for pre- and post-exodontia blood and swabs, respectively). Collectively, we interpreted these results to indicate compositional similarities between the microbial communities present on the gingiva and those detected in post-exodontia blood in a subset of horses, including those whose samples yielded high sequence counts.

**Figure 4:**
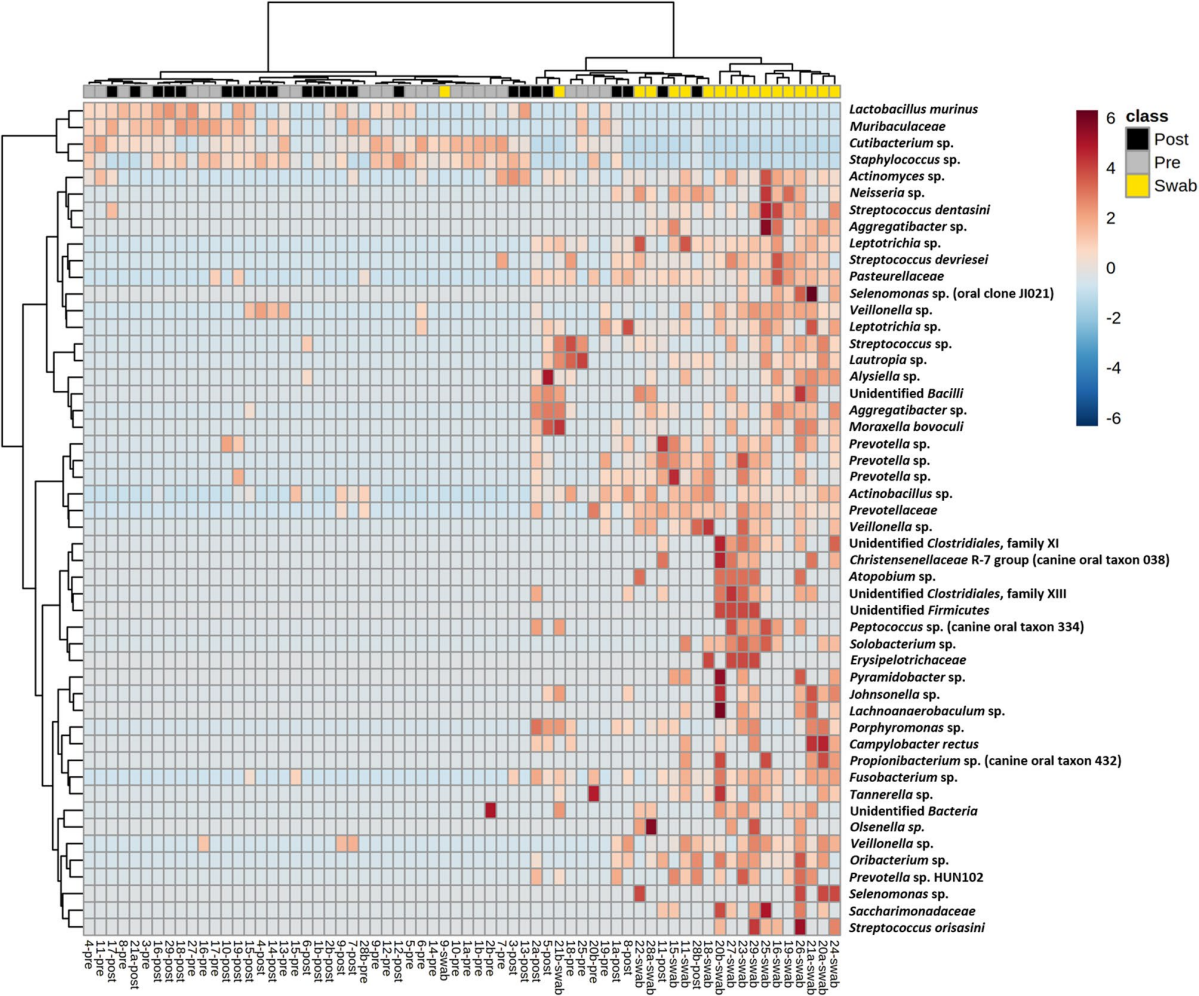
Heatmap generated via hierarchical clustering of samples based on the relative abundance of the 50 ASVs yielding the lowest p values following ANOVA of all ASVs comparing pre- and post- exodontia blood and swabs.

**Figure 5:**
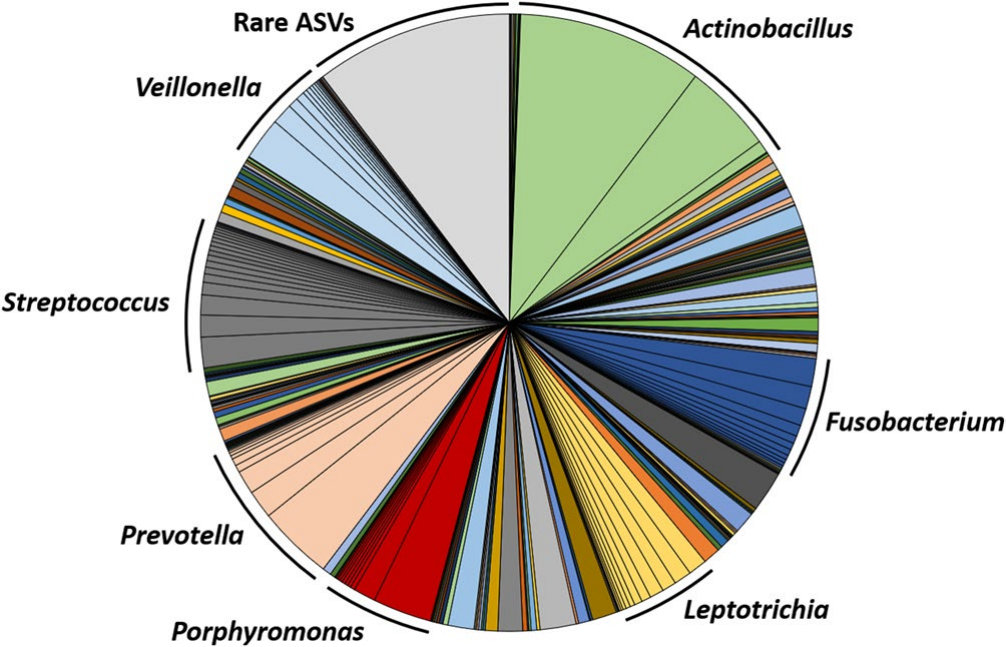
Pie chart showing the mean relative abundance of ASVs detected in the gingival swabs, with dominant genera labeled. The grey portion represents a total of 8544 rare ASVs, comprising roughly 10% of any given sample.

To identify the taxonomies contributing to the differences between swabs and pre- and post-exodontia blood, data from control samples were removed, and serial ANOVA testing was performed on all detected Amplicon Sequence Variants (ASV). Based on those ASVs returning the 50 lowest *p* values, hierarchical clustering was repeated and visualized using a heatmap (Fig. 4). The same post-exodontia blood samples clustered with the gingival swabs, due to the shared presence of multiple taxa associated with the oral cavity including members of the genera *Actinobacillus*, *Fusobacterium*, *Leptotrichia*, *Porphyromonas*, *Prevotella*, *Streptococcus*, and *Veillonella*. Notably, these same taxa linking a subset of post-exodontia blood samples to the gingival microbiota, represent the dominant taxa in the gingival microbiota (Fig. 5). Thus, we interpret the extremely high coverage selectively observed in a subset of post-exodontia samples, and compositional similarities between those samples and the oral cavity microbiota, as compelling evidence of bacteremia resulting from translocated gingival microbiota in horses undergoing exodontia procedures.

## Discussion

To the authors’ knowledge, there have been no previous characterizations of an equine blood microbiome, either in health or disease. The present study is the first to provide information about the equine blood microbiome in adult horses before and after exodontia. 16S rRNA gene profiling has consistently yielded greater microbial diversity in samples with an anticipated low microbial biomass (such as amniotic fluid and blood) than appreciated based on culture-dependent methods^4^. We adopted an approach that had been successfully employed to improve 16S rRNA sequencing in several types of samples, including murine blood^37^. The method entailed increasing the PCR cycle number during library preparation from 25 to 40 cycles and was highly effective in the present study, yielding detection of many ASVs in blood of horses both before and after exodontia. While the requisite reagent controls yielded greater coverage than many of the blood samples, the marked increases observed in a subset of post-exodontia samples, along with the compositional similarity to oral microbiota in those same samples, demonstrate the utility of increased cycle number for similar low microbial biomass samples.

Post-exodontia bacteremia has been well documented in non-equine species and is associated with various potential health complications^4–6,8,10,13,15,17,31,38,39^. Results of earlier studies have varied based on the specific surgical treatment undertaken, method used for bacterial identification, immune system responsiveness, and whether antimicrobial drugs were present in sampled blood at the time of collection^5^. In most instances, distant site bacterial infections (such as bacterial endocarditis) were attributed to bacteria originating from the oral microbiome (including *Streptococcus mitis* and *Streptococcus oralis* in the human medical context)^36^. Although there have been few reports of distant site infections associated with post-exodontia bacterial showering in horses, implicated pathogenic bacteria were also likely derived from oral cavity microbiota^25,26^. Results of the present study show that the 16S rRNA signatures of bacteria present at the gingiva in proximity to an extracted (diseased) tooth are similar to those detected in the blood following exodontia. Moreover, the results of the present study indicate that bacteremia by oral commensal bacteria is still evident one hour following exodontia.

Whereas the human oral microbiome (reportedly the most extensively studied human microflora) has been extensively characterized^36,40^, only a few descriptions of the equine oral microbiome have been published^21,33,41,42^. Approximately 600 prevalent bacterial species have been identified in the human oral cavity based on bacteriological culturing^36^. However, using culture-independent 16S rRNA gene clonal analyses, a majority of bacterial species present in the oral cavity are uncultivable^40,43,44^. Earlier investigations of the equine oral cavity microbiota using bacteriological culturing methods showed that Gram positive cocci (mainly Streptococci, Micrococci, and starch hydrolysers) represent prevalent colonizers in healthy horses^33,45,46^. Both *Gemella* spp. and *Actinobacillus* spp. are also frequently associated with periodontal health in horses^31,33,42^. *Corynebacterium* spp. and *Moraxella* spp. have also been identified in the oral cavity of healthy horses^33^. In another study, *Actinobacillus* spp. and an unclassified *Pasteurellaceae* sp. were the most abundant taxa present in healthy subgingival plaque samples from horses^41^. In that study, *Gammaproteobacteria*, *Firmicutes*, and *Bacteroidetes* (with *Treponema*, *Tannerella*, and *Porphyromonas* species detected at low levels) represented the predominant bacterial phyla identified in the healthy equine subgingival microbiome^21,41^.

16S rRNA gene sequencing was used to show that periodontitis is associated with disruption of the oral cavity microbiota (dysbiosis) in horses^21^. Whereas bacteria in the healthy oral cavity included *Prevotella* spp., *Veillonella* spp., *Gemella* spp., and *Actinobacillus* spp., both *Tannerella* and *Treponema* genera were significantly increased when periodontitis was identified^21^. 16S rRNA PCR was also used to show that acidogenic and aciduric bacteria, including *Streptococcus* species, are associated with peripheral caries in horses, as has been reported in other species^32^. Novel red complex bacteria, *Treponema* and *Tannerella* species, were also identified through their DNA signatures from the gingiva of EOTRH-affected horses^42^. In another study, 18 of 20 horses developed positive blood cultures following exodontia and, in some of those horses, gingival elevation alone resulted in bacteremia^31^. The most commonly identified bacteria on blood culture in that study were *Streptococcus* spp., *Actinomyces* spp., *Fusobacterium* spp., and *Prevotella* spp.; bacterial genera isolated from swab samples of extracted teeth were similar to those detected in the blood, emphasizing that bacteremia resulted from translocation of oral cavity bacteria^31^. However, it should also be noted that results of bacteriological culturing underestimate the extent of bacteremia because most bacteria are uncultivable^34^.

Collectively, these studies demonstrate commonalities in oral microbiota composition between diverse species (human, canine, and feline) and that the equine oral microbiome appears to be broadly similar at the taxonomic level of genus and higher^21,41^. Consistent with previous publications, predominant genera that were identified in the oral cavity of horses in the present study included *Actinobacillus*, *Fusobacterium*, *Leptotrichia*, *Porphyromonas*, *Prevotella*, *Streptococcus*, and *Veillonella*. Moreover, these same taxa were identified in the five post-exodontia blood samples that yielded unexpectedly deep coverage (prolonged bacterial DNA presence). Four of those horses were also affected with sinusitis, suggesting that post-exodontia bacteremia may be more significant when exodontia is undertaken in horses with comorbid sinusitis.

The use of 16S rRNA gene cloning and sequencing methods has led to the discovery that many diverse bacterial phyla that were previously unrecognized or considered unimportant do play a significant role in some diseases^35^. It is becoming increasingly evident that commensal bacteria from the oral cavity microbiome are important in the pathogenesis of post-exodontia complications in people following dental surgery^10,38^. Although 16S rRNA gene cloning and sequencing methods do not differentiate living bacteria from residual bacterial nucleic acid, even residual microbial DNA (in the absence of viable bacterial cells) can serve as an inflammatory signal via innate immune mechanisms including various Toll-like receptors^47^. In light of the fact that a majority of identified bacteria are uncultivable, it is not possible to conclude which, if any, of the identified bacteria are playing a clinically important role in the pathogenesis of exodontia-associated disease based on 16S rRNA signatures^34^.

It has long been recognized that bacteremia resulting from either dental infection or dental surgery can lead to distant infection (such as bacterial endocarditis), especially in immunocompromised individuals^6,14,22–24,30^. Disruption of the gingival-blood barrier as a result of disease or surgical intervention potentially facilitates translocation of bacteria and bacterial products into the circulation, potentially leading to systemic diseases^1^. Moreover, there is emerging realization that anaerobic commensal bacteria from the oral cavity might, given access to the circulation, play a role in the pathogenesis of a remarkable and diverse inventory of extra-oral diseases. Various (human) diseases that have been attributed to this phenomenon include diabetes mellitus, respiratory disease, cardiovascular disease, and atheroma^3,48,49^. Of special interest in this regard is *Fusobacterium nucleatum*, which has been associated with dental disease, various adverse pregnancy outcomes (chorioamnionitis, preterm birth, stillbirth, neonatal sepsis, and preeclampsia), neoplastic and inflammatory gastrointestinal diseases, and various other infections in human patients^3^. Although it remains to be seen whether currently uncultivable oral cavity commensals might contribute to systemic disease in a hitherto unrecognized manner in horses, the fact that periodontal disease is very common in aging horses and that Fusobacteria were prominently identified in post-exodontia blood in the present study suggests that parallel equine studies should be undertaken^50^.

The extent to which post-procedural bacteremia persists has not been extensively reported. In one (human) investigation it was reported that *viridans* group streptococci were rapidly (within 10 min) eliminated from 42 of 46 patients undergoing various oral surgical procedures^5^. In one equine study, two blood samples yielded positive cultures following exodontia (samples obtained 10 min after the termination of surgery), providing evidence for short term persistence of bacteremia^31^. Those authors speculated that persistence of bacteremia could have resulted from a greater number of bacteria (quantitative bacterial counts were not performed) or a result of immune function variations between individual horses (two horses in that study were bacteremic prior to the surgical procedure)^31^. Results of earlier work in other species suggests that intravascular bacteria are rapidly cleared from the circulation by the reticuloendothelial system (within 10–20 min)^51^. Our results show that significant post-exodontia bacteremia is still evident at 60 min following conclusion of surgery in some horses. The immune status of the horses in this study was not examined, but future investigations could incorporate an evaluation of the immune system for horses receiving exodontia surgery. Further studies might also evaluate additional time points beyond one hour for evidence of longer-persisting bacteremia.

The use of prophylactic antimicrobials peri-operatively is restricted to more invasive dental procedures in human dentistry, especially for individuals affected with immunocompromising comorbidities or those with orthopedic implants^52^. Antimicrobials are used under the assumption that they do not prevent bacteremia but inhibit bacterial propagation and bacterial adherence to tissues/implants^52^. Specific guidelines for antimicrobial use in horses receiving exodontia have not been published. Results of the present study showing marked post-exodontia bacteremia persisting for at least one hour suggest that antimicrobial use might be important in this setting, especially for immunocompromised horses.

Using only a solitary time point for blood sampling post-exodontia (one hour post-operatively) was a limitation of this study and the results imply significant post-procedural bacteremia may persist beyond this timeframe and is deserving of further investigation. Although time expended with each exodontia was not measured, it is reasonable to assume that difficult extractions requiring more time could be associated with increased post-procedural bacteremia when compared with more expeditiously concluded procedures. Other limitations include the limited number of cases and the lack of age-matched controls. Blood microbiome results do not necessarily reflect a normal population as all recruited horses were affected with dental disease necessitating exodontia and pre-exodontia blood microbiomes may have been influenced by the presence of dental infection. It should be emphasized that 16S rRNA gene sequencing results are relative, meaning that the actual quantity of bacteria in a given sample is uncertain^53^. It is also possible that each 16S rRNA gene may not amplify with equal efficiency during PCR reactions due to differential primer affinity and GC content and taxonomy assignment is conditional upon the completeness of reference databases^53^. Moreover, multiple studies have demonstrated that increased PCR cycle numbers during library preparation are likely to increase the error rate and introduce bias^37,54,55^. The use of such methods should therefore be based on the sample type and goals of the study, and results interpreted appropriately.

The results of this study affirm that bacteremia resulting from translocated oral cavity commensals occurs in horses following dental extraction. Additionally, post-exodontia bacteremia is still evident in some individuals for up to one-hour, which is much longer than had been previously documented. These results include the first extensive documentation of a blood microbiome based on 16S rRNA gene sequencing in adult horses. The extent of post-exodontia bacteremia, especially as pertains to uncultivable commensal bacteria and their propensity to contribute to extra-oral disease, is deserving of further investigation in horses.

## Materials and methods

### Animals

The study group consisted of 29 adult horses, including 22 geldings and 12 mares, with a mean ± SD age of 19.4 ± 5.6 years (range 3–32 years) and mean ±  SD weight of 479.3 ± 107.1 kg (range 99.0–621.0 kg), presented to the University of Missouri Veterinary Health Center for dental examination and dental extraction. There were a variety of breeds, including 7 Thoroughbreds, 7 American Quarter Horses, 3 American Paint Horses, 2 Hanoverians, and one each of the following breeds: Tennessee Walking Horse, Standardbred, Saddlebred, Oldenburg, American Miniature Horse, Haflinger, National Show Horse, Welsh pony, Missouri Fox Trotting Horse, and Arabian. None of the horses had received antimicrobial drugs for at least 1 week prior to presentation. All horses received both a physical examination and an oral cavity examination. Oral endoscopic and radiographic examinations were used, if indicated.

### Preparation and medication

Horses were placed in stocks. The left jugular vein was subjected to aseptic preparation by clipping and scrubbing with 4% chlorhexidine gluconate that was rinsed using 70% isopropanol. Immediately following skin disinfection, a blood sample (20 mL) was collected from the left jugular vein using a vacutainer needle and immediately transferred into two 10 mL tubes containing ethylenediaminetetraacetic acid (EDTA). Subsequently, an indwelling intravenous catheter was placed into the left jugular vein for drug administration and secured with monofilament suture material. For sedation, horses were given a bolus of detomidine hydrochloride (Orion Pharma Orion Corporation, Espoo, Finland) at 0.01 mg/kg bwt i.v. and butorphanol tartrate (Zoetis Manufacturing and Research, Spain, S.L., Girona, Spain) at 0.01 mg/kg bwt i.v. followed by a constant rate infusion (CRI) of detomidine hydrochloride at 0.005 mg/kg bwt/hr i.v./butorphanol tartrate at 0.005 mg/kg bwt/hr i.v. in saline. Prior to administration of local anesthesia, the gingiva adjacent to both the lingual and buccal aspects of extracted teeth was sampled using a sterile cotton swab that was then placed into a semi‐solid transport medium (Remel, Lenexa, KS, USA). Additionally, anesthesia of the mental, infraorbital, mandibular, or maxillary nerves (as appropriate for location of tooth to be extracted) and local infiltration of the gingiva surrounding the diseased tooth were performed using 2% lidocaine HCl (Hospira, Inc., Lake Forest, IL, USA.). No antimicrobials were given prior to or during extractions.

The oral extraction of cheek, canine, or incisor teeth was performed in a standardized manner as described elsewhere^56–58^. One hour following delivery of the last tooth and cessation of all surgical manipulations, blood was aseptically drawn from the left jugular catheter. The first 10 mL of blood were discarded, and the next 20 mL were collected and transferred into two 10 mL tubes containing EDTA. All blood samples and gingival swabs collected were immediately frozen until further processing. All dental procedures were performed by the same veterinarian. Horse-owners gave informed consent for their animals’ inclusion in this study, which was approved by the institutional Animal Care and Use Committee (MU ACUC# 9233).

### DNA extraction

DNA was extracted from 750 µL whole blood and dental/gingival swabs using PowerFecal kits (Qiagen) according to the manufacturer’s instructions, with the exception that, rather than performing the initial homogenization of samples using the vortex adapter described in the protocol, samples were homogenized in the provided bead tubes using a TissueLyser II (Qiagen, Venlo, Netherlands) for three minutes at 30/second, before proceeding according to the protocol and eluting with 100 µL of elution buffer (Qiagen). DNA yields were quantified via fluorometry (Qubit 2.0, Invitrogen, Carlsbad, CA) using quant-iT BR dsDNA reagent kits (Invitrogen). As negative and positive controls respectively, blank reagents (*n* = 10) and one mock bacterial community standard (ZymoBIOMICS, #D6300) were processed alongside experimental samples.

### 16S rRNA library preparation and sequencing

Extracted blood and gingival swab DNA was processed at the University of Missouri DNA Core Facility. Bacterial 16S rRNA amplicons were constructed via amplification of the V4 region of the 16S rRNA gene with universal primers (U515F/806R) previously developed against the V4 region, flanked by Illumina standard adapter sequences^59,60^. Oligonucleotide sequences are available at proBase^61^. Dual-indexed forward and reverse primers were used in all reactions. PCR was performed in 50 µL reactions containing 100 ng metagenomic DNA, primers (0.2 µM each), dNTPs (200 µM each), and Phusion high-fidelity DNA polymerase (1 U). Amplification parameters were 98 °C^(3 min)^ + [98 °C^(15 s)^ + 50 °C^(30 s)^ + 72 °C^(30 s)^] × 40 cycles + 72 °C^(7 min)^. Amplicon pools (5 µL/reaction) were combined, thoroughly mixed, and then purified by addition of Axygen Axyprep MagPCR clean-up beads to an equal volume of 50 µL of amplicons and incubated for 15 min at room temperature. Products were then washed multiple times with 80% ethanol, and the dried pellet was resuspended in 32.5 µL EB buffer, incubated for 2 min at room temperature, and then placed on the magnetic stand for five minutes. The final amplicon pool was evaluated using the Advanced Analytical Fragment Analyzer automated electrophoresis system, quantified using quant-iT HS dsDNA reagent kits, and diluted according to Illumina’s standard protocol for sequencing on the MiSeq instrument.

### Bioinformatics analysis

The DNA sequences were assembled and annotated at the MU Informatics Research Core Facility. Primers were designed to match the 5′ ends of the forward and reverse reads. Cutadapt (version 2.6; https://github.com/marcelm/cutadapt) was used to remove the primer from the 5′ end of the forward read^62^. If found, the reverse complement of the primer to the reverse read was then removed from the forward read as were all bases downstream. Thus, a forward read could be trimmed at both ends if the insert was shorter than the amplicon length. The same approach was used on the reverse read, but with the primers in the opposite roles. Read pairs were rejected if one read or the other did not match a 5′ primer, and an error-rate of 0.1 was allowed. Two passes were made over each read to ensure removal of the second primer. A minimal overlap of three with the 3′ end of the primer sequence was required for removal.

The Qiime2^63^ DADA2^64^ plugin (version 1.10.0) was used to denoise, de-replicate, and count ASVs, incorporating the following parameters: (1) forward and reverse reads were truncated to 150 bases, (2) forward and reverse reads with number of expected errors higher than 2.0 were discarded, and (3) chimeras were detected using the "consensus" method and removed. R version 3.5.1^65^ and Biom version 2.1.7 were used in Qiime2. Taxonomies were assigned to final sequences using the Silva.v132 database^66^, using the classify-sklearn procedure.

Hierarchical clustering was performed using an unweighted pair group method with arithmetic mean (UPGMA) approach based on unweighted Jaccard similarities. Similarly, principal coordinate analysis was performed using Jaccard similarities. Clustering approaches were executed using Past3 software^67^, downloaded on August 20, 2019.

All methods were carried out in accordance and compliance with relevant guidelines and regulations, including ARRIVE guidelines.

### Statistical analysis

Univariate data were first tested for normality using the Shapiro–Wilk method. Non-normally distributed data were then tested using a Kruskal–Wallis analysis of variance (ANOVA) on ranks, followed by post hoc pairwise comparisons using Dunn’s method, with significance defined by *p* < 0.05. Multivariate data were compared using permutational multivariate ANOVA (PERMANOVA) based on Jaccard similarities, using Past3 software^67^.

### Ethical animal research

MU ACUC# 9233 for opportunistic blood sample acquisition from client owned horses. Horse-owners gave informed consent for their animals’ inclusion in the study.

## Data Availability

The datasets generated and analyzed during the current study are available in the National Center for Biotechnology Information (NCBI) Sequence Read Archive (SRA), as BioProject ID PRJNA674326.
